# Effect of methoxy stilbenes—analogs of resveratrol—on the viability and induction of cell cycle arrest and apoptosis in human myeloid leukemia cells

**DOI:** 10.1007/s11010-020-03837-0

**Published:** 2020-07-31

**Authors:** Małgorzata Zielińska-Przyjemska, Mariusz Kaczmarek, Violetta Krajka-Kuźniak, Marcin Wierzchowski, Wanda Baer-Dubowska

**Affiliations:** 1 Department of Pharmaceutical Biochemistry, Poznan University of Medical Sciences, Poznan, Poland; 2 Department of Clinical Immunology, Poznan University of Medical Sciences, Poznan, Poland; 3 Department of Chemical Technology of Drugs, Poznan University of Medical Sciences, Poznan, Poland

**Keywords:** Methoxy stilbenes, Resveratrol, Apoptosis, Promyelocytic leukemia cells, Monocytic leukemia cells

## Abstract

The present study aimed to evaluate the cytotoxicity and its mechanism of five synthetic methoxy stilbenes, namely 3,4,4ʹ-trimethoxy, 3,4,2ʹ-trimethoxy, 3,4,2ʹ,4ʹ-tetramethoxy, 3,4,2ʹ,6ʹ-tetramethoxy, and 3,4,2ʹ,4ʹ,6ʹ-pentamethoxy-trans-stilbenes (MS), in comparison with resveratrol (RSV). Human promyelocytic (HL-60) and monocytic leukemia (THP-1) cells were treated with the tested compounds for 24 h, and cytotoxicity, cell cycle distribution, and apoptosis were evaluated. Significant differences were found in the susceptibility of these cell lines to all stilbenes, including RSV. The THP-1 cells were more resistant to cytotoxic activity of these compounds than HL-60 cells. Among the tested stilbenes, 3,4,4ʹ-tri-MS and 3,4,2ʹ,4ʹ-tetra-MS exhibited higher cytotoxicity toward both cell lines than RSV and the other methoxy stilbenes. This activity might be related to cell cycle arrest at the G2/M phase and induction of apoptosis. In this regard, 3,4,4ʹ-tri-MS and 3,4,2ʹ,4ʹ-tetra-MS at highest concentrations increased the p53 protein level particularly in HL-60 cells. Moreover, treatment with these derivatives increased the ratio of the proapoptotic Bax protein to the antiapoptotic Bcl-xl protein, suggesting the induction of apoptosis through the intrinsic mitochondrial pathway in both cell lines. Further studies are required to fully elucidate the mechanism of these activities.

## Introduction

Resveratrol (*trans*-3,5,4′-trihydroxystilbene, RSV), a polyphenolic phytoalexin, possesses well-documented cancer chemopreventive and chemotherapeutic activity [1]. RSV has been shown to inhibit in vitro growth of several cancer cells, including human acute lymphocytic and non-lymphocytic leukemia cell lines such as HL-60 (promyelocytic leukemia) [2], K562 (chronic myelogenous leukemia) [3], and CEM-C7H2 (T-acute lymphocytic leukemia) [4]. Several mechanisms have been proposed for the growth inhibitory effect of RSV, including induction of leukemia cell differentiation, apoptosis, and cell cycle arrest at the S phase and inhibition of DNA synthesis by inhibiting ribonucleotide reductase or DNA polymerase [5].

Regarding apoptosis, RSV was shown to induce both extrinsic and intrinsic apoptosis in HL-60 cells. The intrinsic apoptosis pathway is referred to the mitochondrial apoptosis pathway, while extrinsic apoptosis refers to apoptosis initiated by Fas ligands followed by caspase-8 cleavage to an activated form, which subsequently cleaves and activates caspase-3 or induces BID (a BH3 domain-containing proapoptotic Bcl2 family member) cleavage, causing BID to translocate to the mitochondria and induce apoptosis through the intrinsic pathway [6].

RSV, however, has several pharmacokinetic limitations. Particularly, the susceptibility of its hydroxyl groups to glucuronidation and sulfation leads to a short half-life and limited bioavailability. Moreover, the cytotoxic effects of RSV on human cancer cells are observed at relatively high concentrations. For example, its growth inhibitory effects were reported at 40–200 µM [7]. The substitution of the hydroxyl group with methoxy groups enhanced the bioavailability of these derivatives in comparison with that of RSV because of increased lipophilicity and resistance to glucuronidation and sulfation [8].

Compared to RSV, its derivatives with ortho-methoxy substituents such as 3,4,5,4′-tetramethoxy-trans-stilbene (DMU212) have been found to be more potent anticarcinogenic agents in some in vitro and in vivo studies [9, 10].

Moreover, 3,5,4ʹ-trimethoxystilbene (3,5,4ʹ-tri-MS) has emerged as the most potent proapoptotic analog of RSV [11]. In addition, 3,5,4ʹ-tri-MS and pinostilbene (3,4ʹ-dihydroxy-5-methoxystilbene) were reported to be up to 100-fold more cytotoxic than RSV in cancer cell lines [12].

Our previous studies also showed that 3,5,4ʹ-tri-MS caused a massive accumulation of cells at the G2/M phase of the cell cycle and increased the apoptosis rate related to p53 induction in rat C6 glioma cells [13]. In addition, synthetic 3,4,2′-tri-MS, 3,4,2′,4′-tetra-MS, and 3,4,2′,4′,6′-penta-MS modulated the constitutive expression of enzymes and receptors involved in estrogen metabolism in breast normal and cancer cells more efficiently than RSV [14, 15]. Cytochromes P450 1A1 and 1B1 are crucial for the metabolism of estrogen to their reactive electrophilic forms. The most potent inhibitor of CYP1A1 and 1B1 gene expression was 3,4,2′,4′,6′-penta-MS, which reduced the levels of mRNA transcript and protein of both CYPs from 31 to 89% of the initial levels in immortalized cells [14].

These data indicate that the number and position of hydroxyl group substituent in stilbene scaffold might be critical for their biological and anticancer activity.

The present study aimed to evaluate the cytotoxicity and ability of tri-, tetra-, and penta-methoxy stilbenes to induce cell cycle arrest and apoptosis in human myeloid leukemia cells.

## Materials and methods

### Chemicals

**Fig. 1:**
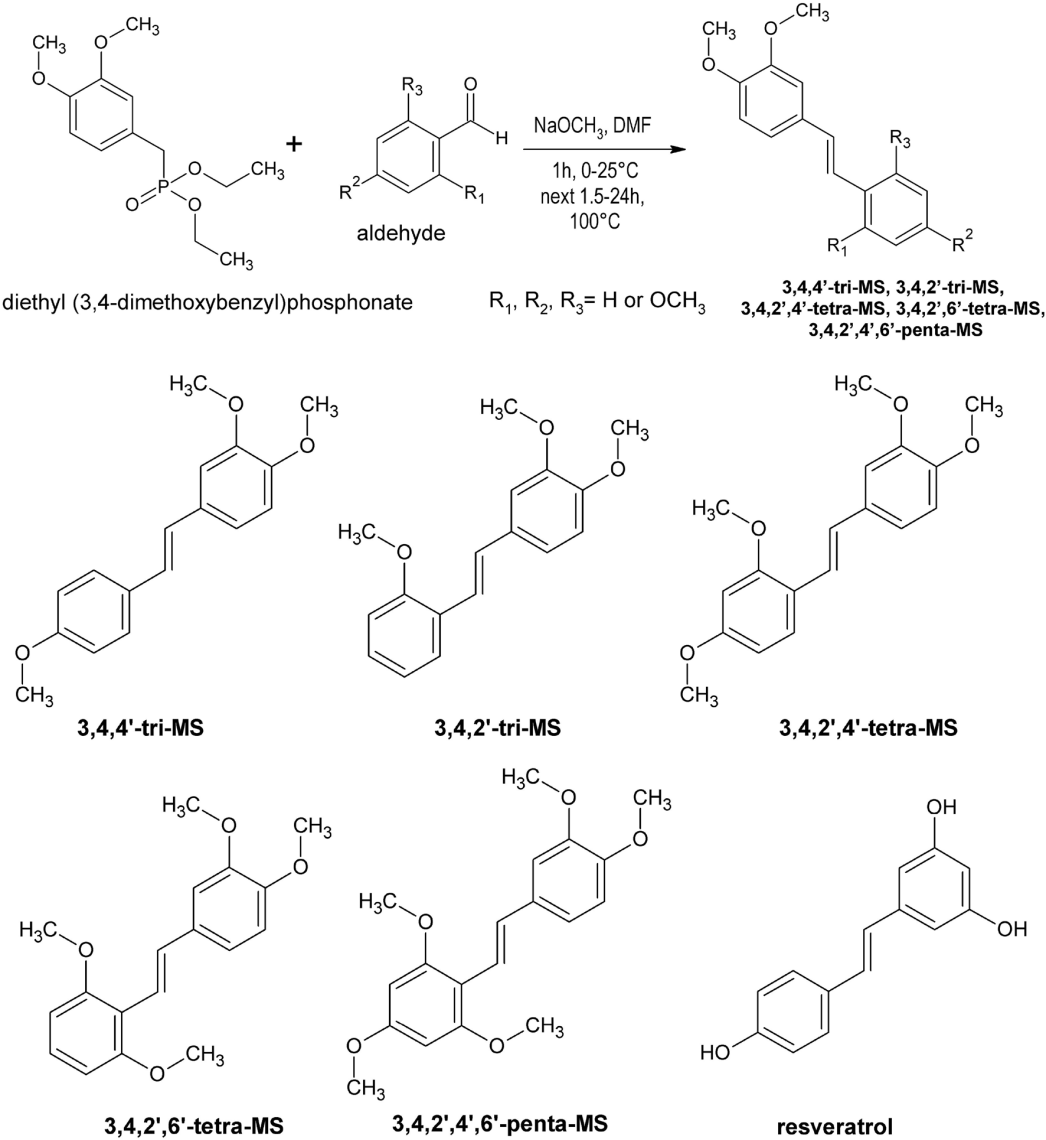
A scheme of methoxy stilbenes (MS) synthesis and their structures

Methoxy stilbenes, namely 3,4,4ʹ-tri-MS, 3,4,2ʹ-tri-MS, 3,4,2ʹ,4ʹ-tetra-MS, 3,4,2ʹ,6ʹ-tetra-MS, 3,4,2ʹ,4ʹ,6ʹ-penta-MS, were synthesized in the Department of Chemical Technology of Drugs, PUMS, as described previously [16]. Diethyl (3,4-dimethoxybenzyl) phosphonate was transformed by the Horner–Wadsworth–Emmons reaction with proper aromatic aldehydes (2-methoxybenzaldehyde or 2,4-dimethoxybenzaldehyde or 2,4,6-trimethoxybenzaldehyde) into the investigated polymethoxy-*trans*-stilbenes. The structures of all compounds were confirmed by NMR, mass spectra, and elementary analysis. Figure 1 outlines the synthesis of these compounds.

RSV, camptothecin, antibiotic solution (10,000 units penicillin, 10 mg streptomycin, and 25 μg amphotericin B per mL), dimethyl sulfoxide (DMSO), fetal bovine serum (FBS), glutamine, propidium iodide (PI), ribonuclease A (RNase A), 3-(4,5-dimethylthiazol-2-yl)-2,5-diphenyltetrazolium bromide (MTT), and RPMI 1640 medium were provided by Sigma-Aldrich Co. (St. Louis, MO, USA). Suppliers of antibodies and reagent kits used in this study are indicated in the description of the respective methods.

All other compounds were readily available commercial products. RSV and its methoxy derivatives were dissolved in DMSO at the concentration of 10 mM and stored at − 20 °C.

### Cell cultures and treatments

HL-60 (human acute promyelocytic leukemia) and THP-1 (human acute monocytic leukemia) cell lines were obtained from the ECACC and supplied by Sigma-Aldrich. HL-60 and THP-1 cells were cultured in RPMI 1640 media supplemented with 10% FBS, antibiotics, and 2 mM l-glutamine in a fully humidified atmosphere at 37 °C with 5% CO_2_. The cells (1 × 10^6^/well) were seeded in 6-well plates by resuspension in complete growth media. The cells were then treated with increasing concentrations of methoxy stilbenes and RSV (1–150 µM). The incubation was continued for subsequent 24 h to assess cell cycle distribution, apoptosis, and the levels of p53, Bax, and Bcl-xl proteins.

Control cells were treated with vehicle (DMSO). Cells treated with 50 nM of camptothecin were used as a positive control. The concentration of DMSO in culture medium did not exceed 0.1%.

### Cell viability (MTT) assay

HL-60 and THP-1 cells were seeded at 5 × 10^4^ cells/well in a 24-well plate. The cells were incubated in triplicates with different concentrations of methoxy stilbenes or RSV (1–200 μM) in a final volume of 100 µL of growth medium for 24 and 48 h at 37 °C with 5% CO_2_. An aliquot of 10 µL of MTT solution (5 mg/mL) was added to each well and incubated for another 4 h. The water-insoluble formazan crystals were solubilized in DMSO before the measurement of absorbance using a microplate reader (TECAN Infinite M200) at 570 nm (with the reference wavelength at 690 nm). All the experiments were repeated three times, with at least three measurements per assay.

### Flow cytometric cell cycle analysis

Cell cycle distribution was evaluated by flow cytometric analysis using PI staining. After treatment, HL-60 and THP-1 cells were collected, washed with PBS, and fixed in 70% ethanol at 4 °C for 30 min. The cells were then washed twice in PBS and resuspended in 250 µL of PBS containing 50 µg/mL PI and 100 µg/mL RNase A. After incubation in dark at 37 °C for 30 min, the fluorescence of cells was measured with a FACSCanto flow cytometer (Becton Dickinson, San Jose, CA, USA). Data analysis and acquisition were performed using FACS Diva software (Becton Dickinson).

### FITC Annexin V/PI double staining assay

Apoptosis was determined by Annexin V and PI double staining of treated and non-treated HL-60 and THP-1 cells by using the FITC Annexin V Apoptosis Detection kit (Becton Dickinson) following the manufacturer’s procedures. Briefly, harvested cells were washed with PBS, resuspended in 100 μL of binding buffer, and stained with 5 μL of FITC Annexin V labeling reagent and 5 μL of PI for 15 min in dark. Finally, the samples were assessed with a FACSCanto flow cytometer using Cell Quest software. In our study, apoptotic cells included cells with early (Annexin V+, PI−) and late (Annexin V+, PI+) apoptosis, while viable cells were negative for both Annexin V and PI.

### Western blot analysis of p53, Bax, and Bcl-xl protein levels

The immunoblot assay was used to determine the level of p53, Bax, and Bcl-xl proteins. Briefly, after 24 h culture with various concentrations of methoxy stilbenes or RSV, cell lysates were obtained by extracting HL-60 and THP-1 cells with RIPA buffer. The concentration of total proteins was determined using the Bradford protein assay (Bio-Rad Laboratories, Hercules, CA, USA). Fifty micrograms of total protein from each concentration was resolved by electrophoresis using 12% or 10% SDS-PAGE, followed by electrotransfer onto a polyvinyl difluoride transfer membrane (Immobilon-P, Millipore). After blocking with 10% skimmed milk, the proteins were probed with anti-human p53, Bax, and Bcl-xl antibodies. The β-actin protein was used as an internal control. As the secondary antibodies in the staining reaction, alkaline phosphatase-labeled anti-goat IgG, anti-mouse IgG, or anti-rabbit IgG was used. Bands were visualized with the Bio-Rad AP Conjugate Substrate Kit NBT/BCIP. The amount of the immunoreactive product in each lane was determined using the Quantity One software (Bio-Rad Laboratories). Values were calculated as relative absorbance units (RQ) per mg protein.

### Statistical analysis

All results are presented as mean ± SEM from at least three independent experiments. Statistical evaluation was performed with one-way ANOVA and Dunnett’s post hoc test using GraphPad Prism Software Version 4.03 (San Diego, CA, USA). Differences were considered to be significant for *p* values ≤ 0.05.

## Results

### Effect of RSV and methoxy stilbenes on cell viability

The effect of the tested methoxy stilbenes and RSV on the viability of HL-60 and THP-1 cells was assessed using the MTT assay*.* Table 1 presents the IC_50_ values obtained after 24 and 48 h of incubation with the tested compounds. Significant differences were noted in the effect of these compounds on the viability of HL-60 and THP-1 cells. The IC_50_ values in HL-60 cells were approximately two times lower than those obtained in THP-1 cells. Moreover, the cytotoxicity of RSV in comparison with its methoxy analogs in HL-60 cells, particularly after 48 h treatment, did not differ significantly. In contrast, THP-1 cells were more susceptible to the cytotoxic effect of RSV analogs than to the parent compound. The highest cytotoxicity was shown by 3,4,4ʹ-tri-MS followed by 3,4,2ʹ,4ʹ-tetra-MS.

**Table 1:** Effect of resveratrol and its derivatives on human HL-60 and THP-1 cell viability (IC50; μM)

Tested compounds	IC50 (μM)			
	HL-60		THP-1	
	24 h	48 h	24 h	48 h
3,5,4′-trihydroxy-*trans-*stilbene (Resveratrol)	59.98 ± 1.54	46.84 ± 1.92	130.05 ± 1.37^#^	79.02 ± 1.63^##^
3,4,4′-trimethoxy-*trans-*stilbene (3,4,4′-tri-MS)	51.36 ± 2.09*	41.32 ± 1.70	97.25 ± 3.11*,^#^	62.81 ± 1.83*,^##^
3,4,2′-trimethoxy-*trans-*stilbene(3,4,2′-tri-MS)	58.27 ± 1.47	46.34 ± 1.89	121.41 ± 2.08^#^	75.20 ± 2.64^##^
3,4,2′,4′-tetramethoxy-*trans-*stilbene (3,4,2′,4′-tetra-MS)	55.35 ± 2.23	43.01 ± 2.68	117.35 ± 3.68*,^#^	70.48 ± 2.45^##^
3,4,2′,6′-tetramethoxy-*trans-*stilbene (3,4,2′,6′-tetra-MS)	61.32 ± 3.80	40.00 ± 3.04	127.60 ± 3.24^#^	77.26 ± 3.17^##^
3,4,2′,4′,6′-pentamethoxy-*trans-*stilbene (3,4,2′4′,6′-penta-MS)	56.85 ± 1.17	44.52 ± 2.40	118.52 ± 3.05^#^	77.71 ± 1.91^##^

The cells were treated for 24 and 48 h with different concentrations of methoxy stilbenes or resveratrol and IC50 values were obtained by plotting log (% inhibition/100 − % inhibition) vs log (concentration), where % inhibition = (100 − viability) based on means ± SEM from three independent experiments

*p < 0.05 compared to resveratrol treated cells; ^#^*p* < 0.05 compared to HL-60 cells treated for 24 h; ^##^*p* < 0.05 compared to HL-60 cells treated for 48 h

### The effect of RSV and methoxy stilbenes on cell cycle distribution

**Fig. 2:**
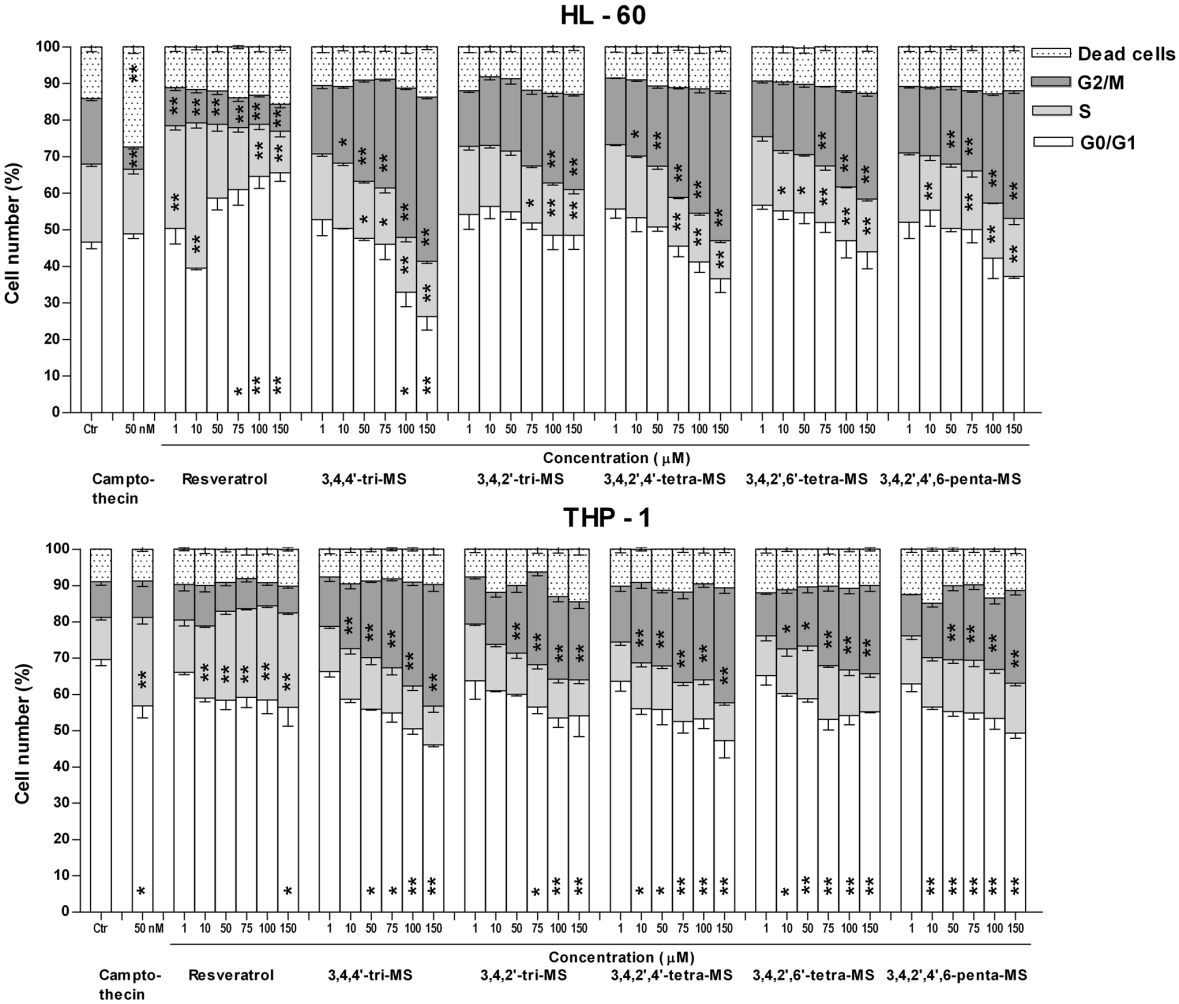
Cell cycle distribution in human myeloid leukemia cells after 24 h of incubation with resveratrol and methoxy stilbenes followed by propidium iodide labeling and flow cytometry analysis. Camptothecin at the final concentration of 50 nM was used as a positive control. Results of three independent experiments are presented as mean ± SEM. *p < 0.05, **p < 0.01 compared to untreated control using ANOVA followed by Dunnett’s test

To assess the effect of RSV and methoxy stilbenes on cell cycle distribution, the HL-60 and THP-1 cells were treated with these compounds for 24 h, and the cell cycle progression was assessed by PI staining of nuclear DNA using flow cytometry. Camptothecin, the DNA topoisomerase I inhibitor, at a final concentration of 50 nM was used as a positive control. Figure 2 presents the results of this assay.

A significant increase in the number of THP-1 cells in the S phase as a result of camptothecin treatment was observed; this finding is consistent with its described ability to arrest cell cycle in this phase. However, camptothecin treatment of HL-60 myeloid cells led to an increased number of dead cells and reduced the percentage of HL-60 cells in the S phase.

Consistent with the cytotoxicity data, 3,4,4ʹ-tri-MS and 3,4,2ʹ,4ʹ-tetra-MS increased mostly the percentage of cells in the G2/M phase in HL-60 and THP-1 cells. A similar effect was observed after treatment with other methoxy stilbenes, but the percentage of cells in this phase was lower than that observed after treatment with 3,4,4ʹ-tri-MS and 3,4,2ʹ,4ʹ-tetra-MS. In contrast, RSV increased the number of cells in the S phase in THP-1 cells. In HL-60 cells, RSV tended to increase the percentage of cells in the S phase after treatment with lower concentrations (1–10 µM), while at higher concentrations (75–150 µM), a considerable accumulation of cells in the G0/G1 phase was observed.

### The effect of RSV and methoxy stilbenes on the induction of apoptosis

**Fig. 3:**
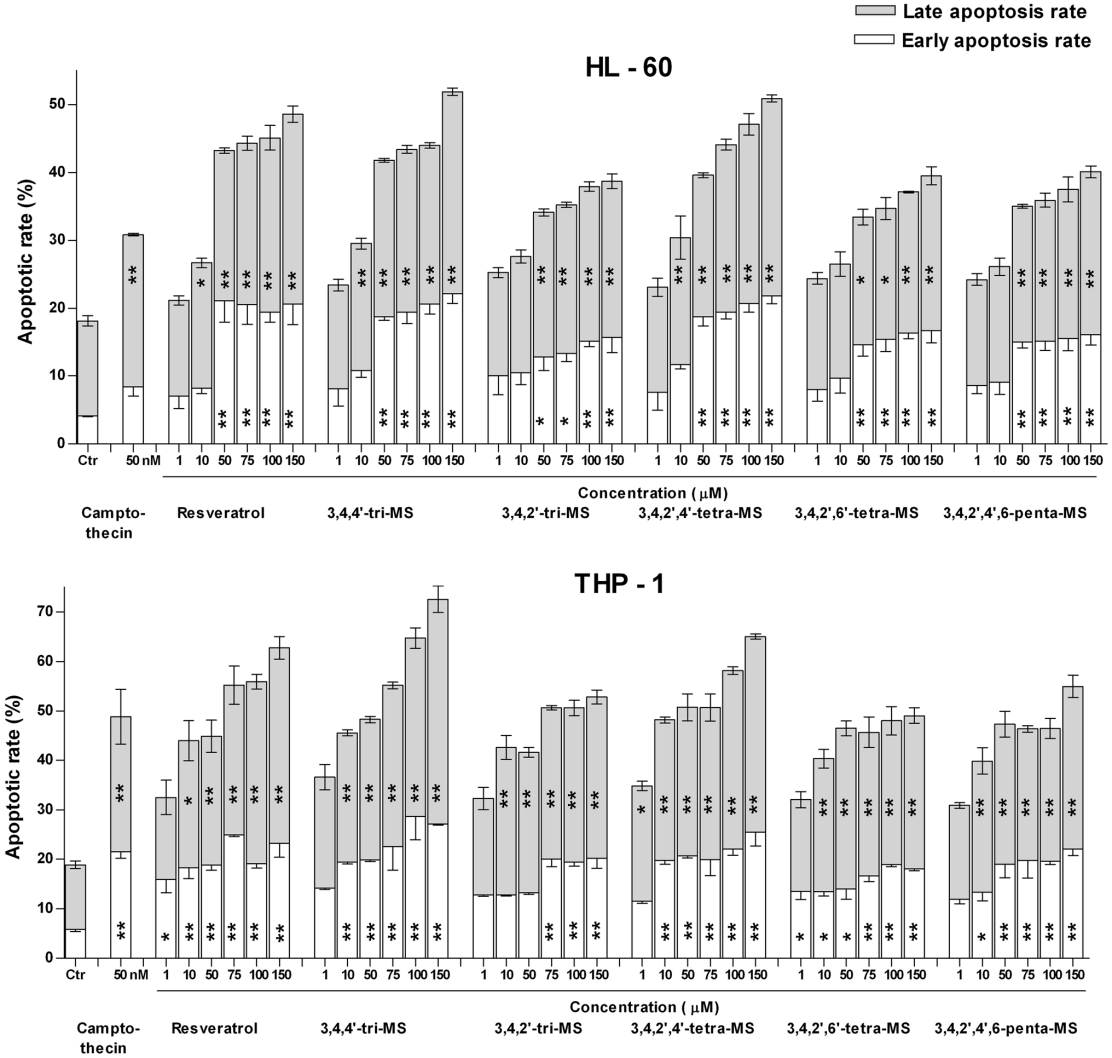
Phosphatidylserine externalization measured by Annexin V and PI double staining in human myeloid leukemia cells after 24 h of incubation with resveratrol and methoxy stilbenes. Camptothecin at the final concentration of 50 nM was used as a positive control. Results of three independent experiments are presented as mean ± SEM. *p < 0.05, **p < 0.01 compared to untreated control using ANOVA followed by Dunnett’s test

Figure 3 shows the effect of RSV and its methoxy derivatives on the induction of apoptosis as assessed by Annexin V/PI staining. Treatment with all stilbenes induced apoptosis in both cell lines, particularly at their late stage.

The apoptotic rate was higher in THP-1 cells, and 3,4,4ʹ-tri-MS was the most efficient inducer of this process. The same compound also seemed to be the most efficient apoptosis inducer in HL-60 cells, but only slightly exceeded RSV and 3,4,2ʹ,4ʹ-tetra-MS.

### The effect of RSV and methoxy stilbenes on the p53, Bax, and Bcl-xl protein levels

**Fig. 4:**
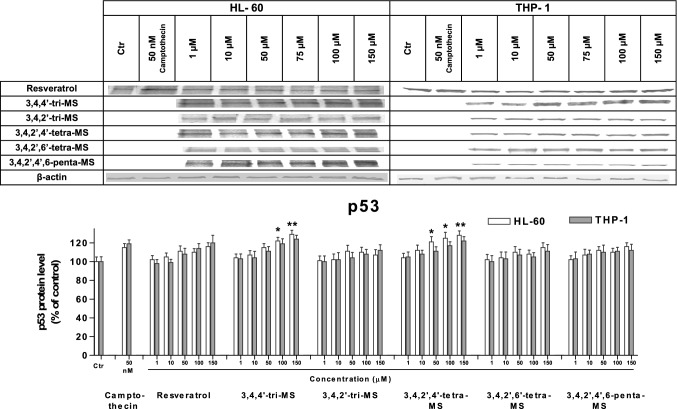
The p53 protein level in HL-60 and THP-1 cells treated with resveratrol and methoxy stilbenes, as determined by Western blot analysis. Results of three independent experiments are presented as mean ± SEM. *p < 0.05 compared to untreated control

**Fig. 5:**
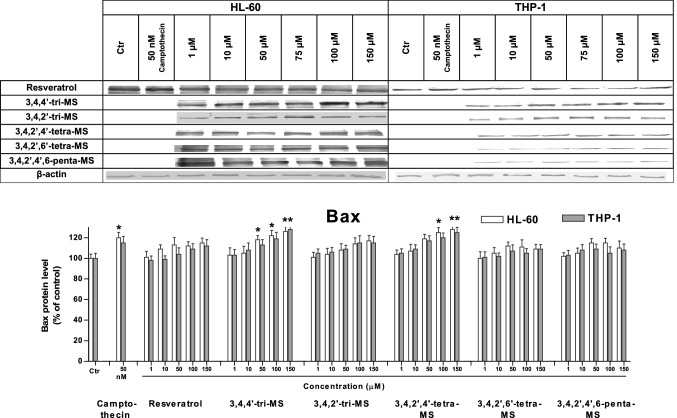
The Bax protein level in HL-60 and THP-1 cells treated with resveratrol and methoxy stilbenes, as determined by Western blot analysis. Results of three independent experiments are presented as mean ± SEM. *p < 0.05 compared to untreated control

**Fig. 6:**
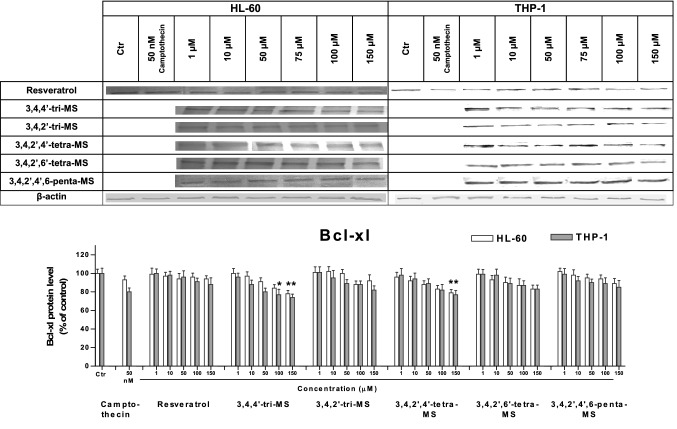
The Bcl-xl protein level in HL-60 and THP-1 cells treated with resveratrol and methoxy stilbenes, as determined by Western blot analysis. Results of three independent experiments are presented as mean ± SEM. *p < 0.05 compared to untreated control

To understand the mechanism of stilbene-induced apoptosis, the levels of the proapoptotic protein Bax and the antiapoptotic protein Bcl-xl along with p53 were evaluated. The results are shown in Figs. 4, 5, 6.

The p53 protein level was increased in both cell lines after treatment with 3,4,4ʹ-tri-MS and 3,4,2ʹ,4ʹ-tetra-MS at the concentration of 150 µM. At lower concentrations (50 and 100 µM), an increased p53 protein level was found in only HL-60 cells. The treatment with these derivatives increased the ratio of proapoptotic Bax to antiapoptotic Bcl-xl.

## Discussion

The present study evaluated the cytotoxic effect of five methoxy analogs of RSV differing in the number and position of substituted hydroxyl groups on human promyelocytic cells HL-60 and human monocytic leukemia cells THP-1. Significant differences were found in the susceptibility of these cell lines to all stilbenes, including RSV. THP-1 cells were more resistant to cytotoxic activity of these compounds than HL-60 cells. The cytotoxicity of RSV and its possible mechanism has been the subject of several studies, together with the cells used in our current investigation. Interestingly, an early study by Ferry-Dumazet et al. [2] showed an opposite effect of RSV on these cell lines. THP-1 cells showed higher sensitivity to RSV than HL-60 cells. The reasons for this discrepancy might be the longer time of exposure (72 h) and the different cytotoxicity assay applied in the above study. In contrast to the MTT assay used in our study, trypan blue exclusion test measures dead cells, while the MTT assay measures viable and proliferating cells. On the other hand, comparable value of IC_50_ value for RSV in HL-60 cells was found by Li et al. [17].

Among the methoxy stilbenes, 3,4,4ʹ-tri-MS showed the highest cytotoxicity, followed by 3,4,2ʹ,4ʹ-tetra-MS with the lowest IC_50_ values in both cell lines. In our previous study, naturally occurring tri-MS substituted in the positions 3,5,4ʹ was also more cytotoxic (by ~ 40–50%) than RSV and pterostilbene (3,5-dimethoxy-4-hydroxystilbene) in C6 rat glioma cells, but in much less extent in human T98G glioma cells [13].

Our recent study (unpublished data) showed that among the RSV analogs evaluated, 3,4,2ʹ,4ʹ-tetra-MS showed the highest cytotoxicity, while 3,4,2ʹ,6ʹ-tetra-MS showed the lowest toxicity in T98G glioma cells.

These observations suggest that not only the number but also the positions of methoxy groups in stilbene rings influence cytotoxicity. Moreover, similar to that of RSV, the cytotoxicity of methoxy stilbenes is clearly cell-specific. In this regard, Takashina et al. [18] demonstrated that RSV is more cytotoxic and can induce apoptotic cell death in human leukemic cells to a greater extent than in human solid tumor cells. Here, we found differences between leukemia cells of different origin in the susceptibility to the tested stilbenes.

Similar differences were observed in the effect of the tested stilbenes on cell cycle distribution. Moreover, camptothecin, which was used as a positive control, also differently affected cell cycle distribution in these two leukemic cell lines. This topoisomerase I inhibitor is known to arrest cell cycle in the S phase [19]. This effect was observed in THP-1 cells, but in HL-60 cells, a significant increase in the number of dead cells was observed after camptothecin treatment. This finding suggests that the latter cells are also more sensitive to camptothecin. As previously reported by us and other authors, RSV increased the percentage of cells in the S phase [13, 20–23]. Interestingly, in HL-60 cells, this effect was mainly seen after treatment with the lowest concentrations of RSV. This effect may be related to hormesis shown by RSV in some systems [24].

Methoxy stilbenes tended to enhance the percentage of cells in the G2/M phase. In both cell lines, a clear concentration-dependent relationship was observed, with the most significant increase after treatment with 3,4,4ʹ-tri-MS and 3,4,2ʹ,4ʹ-tetra-MS, thus in agreement with their highest cytotoxicity. Consequently, the increased rate of apoptosis, particularly a higher percentage of late apoptotic cells, was observed in THP-1 cells after treatment with all stilbenes, but the highest effect was observed after treatment with 3,4,4ʹ-tri-MS.

An earlier study by Traversi et al. [25] showed that the most extensively studied 3,5,4ʹ-tri-MS, in contrast to RSV that delays proliferation of cancer cells during the S phase, causes cell cycle arrest at the G2/M phase, thus showing a different way of delaying cell cycle progression. Therefore, the results of the present study confirm a similar effect for 3,4,4ʹ-tri-MS. Moreover, this group provided evidence that the substitution of hydroxyl groups with methoxy ones renders the interaction between the RSV derivatives with DNA and topoisomerase II much weaker due to the reduced numbers of hydrogen bonds between the methoxy derivatives, topoisomerase II, and DNA. It is possible that the most active methoxy stilbenes evaluated in our study act in a similar way, but further studies are required to confirm this possibility.

To determine the possible mechanism of apoptosis induction, the protein levels of p53, Bax, and Bcl-xl were assessed. The p53 pathway has been shown to mediate cellular stress responses as p53 can initiate DNA repair, cell cycle arrest, and apoptosis [26]. The latter is activated to eliminate cells with severe DNA damage and thereby inhibit the transfer of damaged DNA to daughter cells [27]. This mechanism may be involved in the proapoptotic activity of 3,4,4ʹ-tri-MS and 3,4,2ʹ,4ʹ-tetra-MS, which at highest concentrations (100 and 150 µM for tri-MS and 50–150 µM for tetra-MS) increased the p53 protein level, particularly in HL-60 cells.

Moreover, treatment with these derivatives increased the ratio of the proapoptotic protein Bax to the antiapoptotic protein Bcl-xl. This effect might be related to the increased level of p53, which controls the transcription of these proteins [28]. This finding suggests that 3,4,4ʹ-tri-MS and 3,4,2ʹ,4ʹ-tetra-MS induce apoptosis through the intrinsic mitochondria pathway in both cell lines.

To summarize, our study showed that 3,4,4ʹ-tri-MS and 3,4,2ʹ,4ʹ-tetra-MS exhibited higher cytotoxicity toward human promyelocytic (HL-60) and monocytic leukemia (THP-1) cells than RSV and other methoxy stilbenes. This activity might be related to cell cycle arrest at the G2/M phase and induction of apoptosis. Further studies, which will include the detailed analysis of cell cycle and apoptosis regulating proteins, are required to fully elucidate the mechanism of these activities.

## Abbreviations

DMSO: Dimethyl sulfoxide
FBS: Fetal bovine serum
MTT: 3-(4,5-Dimethylthiazol-2-yl)-2,5-diphenyltetrazolium bromide
PI: Propidium iodide
PS: Phosphatidylserine
RNase A: Ribonuclease A
